# *Gpr174-*deficient regulatory T cells decrease cytokine storm in septic mice

**DOI:** 10.1038/s41419-019-1462-z

**Published:** 2019-03-08

**Authors:** Dongze Qiu, Xun Chu, Laiqing Hua, Yunke Yang, Keyong Li, Yi Han, Jun Yin, Ming Zhu, Sucheng Mu, Zhan Sun, Chaoyang Tong, Zhenju Song

**Affiliations:** 1 Department of Emergency Medicine, Zhongshan Hospital, Fudan University, Shanghai, China; 2 Department of Integrative Medicine, Zhongshan Hospital, Fudan University, Shanghai, China; 3 Xinhua Hospital, Shanghai Institute for Pediatric Research, Shanghai Jiao Tong University School of Medicine, Shanghai, China; 4 Department of Genetics, Shanghai-MOST Key Laboratory of Health and Disease Genomics, Chinese National Human Genome Center, Shanghai, China; 5 Affiliated Cancer Hospital & Institute of Guangzhou Medical University, Guangzhou, China; 6 Department of Pharmacology, University of Virginia School of Medicine, Charlottesville, VA USA

## Abstract

*G protein-coupled receptor 174* (*GPR174*) is mainly expressed in thymus, spleen, lymph nodes, and leukocytes, and genetic variation in *GPR174* is associated with susceptibility to autoimmune diseases, indicating that GPR174 is involved in the immune response. However, the function of GPR174 in regulating inflammatory responses against bacterial infection in sepsis remains unclear. In this study, we investigated the role of GPR174 in regulating suppressive function of regulatory T cells (Treg cells) and the underlying mechanism of *Gpr174*-deficient Treg cells in controlling cytokine storm of sepsis. We showed that *Gpr174*-dedicient mice were resistant to inflammatory shock induced by lipopolysaccharide (LPS) and cecal ligation and puncture (CLP). Moreover, *Gpr174* was highly expressed in Treg cells, and its deficiency in mice promoted the expression of cytotoxic T lymphocyte associated antigen 4 (CTLA-4) and interleukin (IL)−10 in Treg cells. By using the LPS-induced sepsis model, we demonstrated that anti-inflammatory macrophages (M2 macrophages) induction was Treg cell-dependent and *Gpr174*-deficient Treg cells protected mice against sepsis-induced lung damage through prompting M2 macrophages polarization. In vitro, *Gpr174*-deficient Treg cells also promoted the polarization of macrophages toward M2 cells and dampened the secretions of pro-inflammatory cytokines (IL-6 and tumor necrosis factor-α (TNF-α)) in macrophages. In conclusion, these findings suggested that GPR174 plays an important role in the initial period of sepsis through the regulation of macrophage polarization and pro- and anti-inflammatory cytokine secretions. Therefore, GPR174 may be a promising target for therapeutic agents to regulate inflammatory disorders.

## Introduction

Sepsis is a life-threatening syndrome of organ dysfunction induced by the dysregulation of host immune responses to infection^1^. Although the hospital supportive cares have been improved, sepsis is still the major cause of mortality in intensive care unit^2^. Both innate and adaptive immune cells mediate the overwhelming inflammatory response of sepsis^3–5^. The innate immune cells, such as neutrophils, macrophages, dendritic cells, and nature killer T cells, play pivotal roles in the systemic inflammatory response involved in the development of sepsis and sepsis-induced organ injury. Recent studies revealed that Treg cells not only play an indispensable role in prevention of the occurrence of autoimmune diseases, allergies, and transplant rejection, but also control innate immune activation in response to LPS-challenge in sepsis^6–8^.

*GPR174* variation has been proposed to be a risk factor for Graves’s disease^9^, autoimmune Addison’s disease^10^ and vasovagal syncope^11^. These results indicate that the GPR174 plays an important role in immune response. However, the role of GPR174 in the immune response of sepsis is unclear. GPR174 is a G protein-coupled receptor (GPCR) and belongs to P2Y receptor family and is highly expressed in thymus, spleen, and lymph node^12^. GPR174 is activated by lysophosphatidylserine (LysoPS)^13^, a lipid mediator known to induce rapid degranulation of mast cells^14,15^, suppress proliferation of T lymphocytes^16^ and enhance macrophage phagocytosis of apoptotic neutrophils^17,18^. A recent study showed that GPR174 have a negative role in the development and function of Treg cells^19^.

In the present study, we investigated whether GPR174 played a role in the process of sepsis via regulation of Treg cells function. Firstly, we generated *Gpr174* knockout (KO) mice and found that depletion of *Gpr174* resulted in higher expressions of IL-10 and CTLA-4 in Treg cells. Furthermore, we found that depletion of *Gpr174* alleviated the tissue damage and promoted the polarization of macrophages toward M2-like cells induced by sepsis via Treg cells. Meanwhile, the suppressive function of Treg cells on the secretion of IL-6 and TNF-α of macrophages was enhanced in *Gpr174*-deficient mice compared to wide type (WT) mice. Taken together, these results suggested that GPR174 affects the sepsis pathogenesis *via* regulation of Treg cells suppressive functions.

## Results

### *Gpr174* KO mice were resistant to inflammatory shock induced by LPS and CLP

**Fig. 1:**
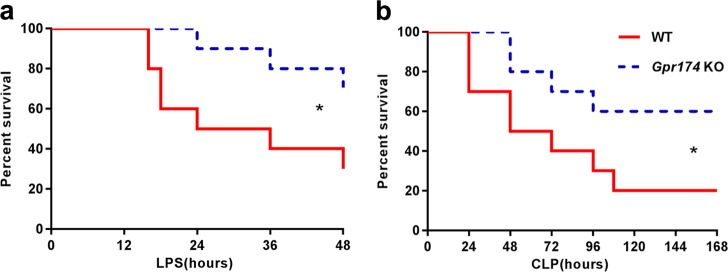
*Gpr174* KO mice were resistant to LPS-induced endotoxemia and CLP -induced sepsis. **a** WT and *Gpr174* KO (*n* = 20 mice/group) mice were injected (intraperitoneal injection, i.p.) with LPS (10 mg/kg). Survival rate was monitored for 48 h. **b** WT and *Gpr174* KO (*n* = 20 mice/group) mice were subjected to CLP. Survival rate was monitored for 168 h. Mortality rates were compared using Kaplan–Meir method with Log-rank Test. *P < 0.05

To explore the function of GPR174 in the development of sepsis, we generated a mouse model with global-targeted deletion of *Gpr174* (Supplementary Fig. 1). *Gpr174* KO mice were viable and could reach old age (12 months) without any gross development abnormalities. To determine whether GPR174 plays a role in the pathogenesis of sepsis, we produced LPS-induced endotoxic shock model using *Gpr174* KO and WT mice respectively (*n* = 20 per group), and monitored survivals for 48 h. *Gpr174* KO mice were resistant to LPS with a survival rate of 70%, whereas WT mice were sensitive to endotoxic shock with a survival rate of 30% (*P* = 0.045, Fig. 1a). To further determine whether this observation could be replicated in a clinically relevant sepsis model, we induced polymicrobial sepsis by CLP as described previously^20^. It resulted in a worse phenotype in WT mice at the end of CLP experiment (168 h), only 20% of WT mice survived, however, 60% of *Gpr174* KO mice survived (*n* = 20 per group, *P* = 0.037, Fig. 1b).

### Alterations of regulatory molecules in Treg cells from *Gpr174*-deficient mice

It was reported that GPR174 limited the suppressive function of mouse Treg cells^19^, but the mechanism is not clear. To investigate the influence of GPR174 on function of Treg cells, we first measured the expression level of *Gpr174* mRNA in Treg cells from splenocytes of WT mice. *Gpr174* mRNA was mainly expressed in Treg cells (CD4^+^CD25^+^T cells), CD4^+^ T cells, CD8^+^ T cells and B cells, whereas it was expressed at a low level in macrophages (Supplementary Fig. 2).

**Fig. 2:**
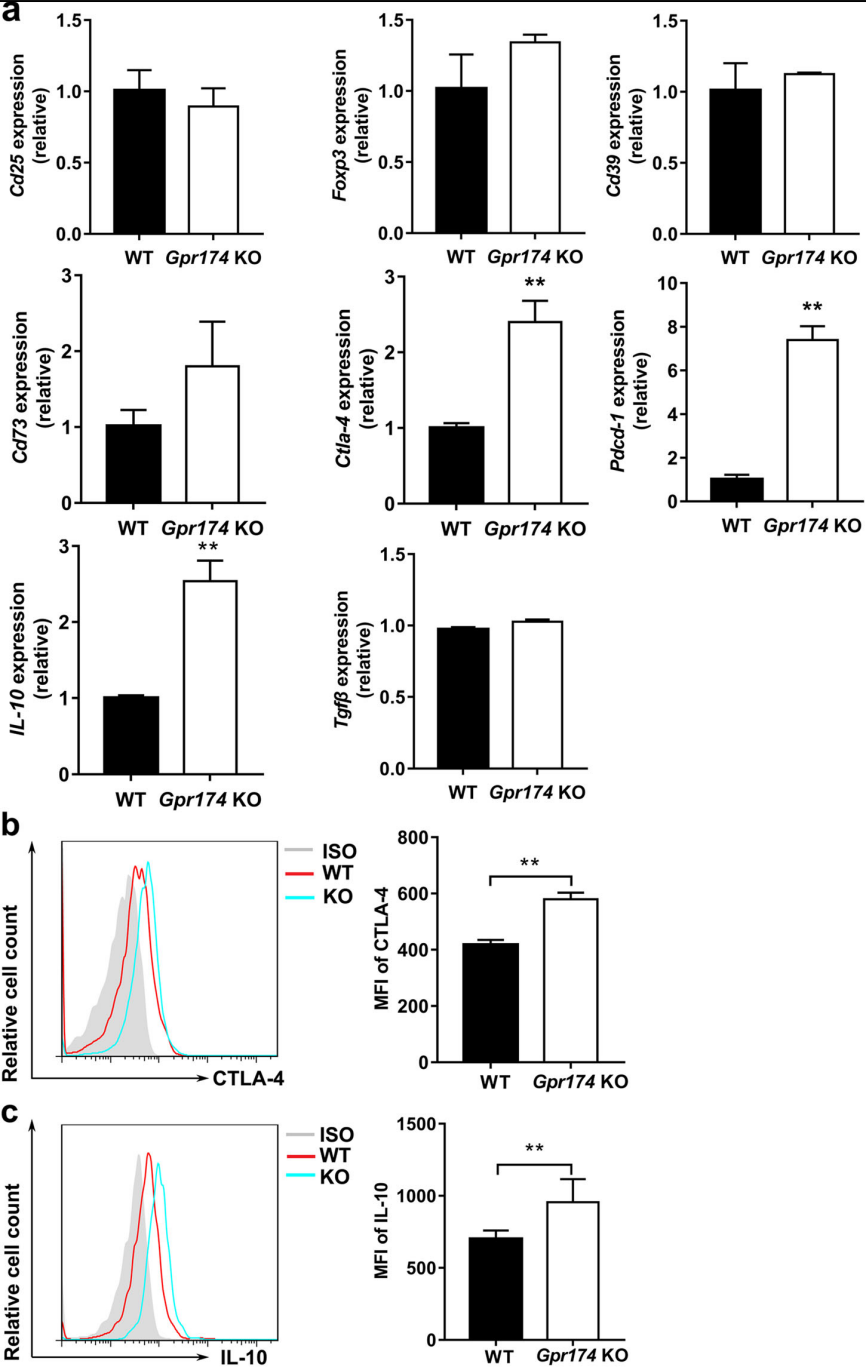
Altered suppressive function related markers of *Gpr174*-deficient Treg cells. **a** Comparison of mRNA levels between Treg cells in spleens of WT and *Gpr174*-deficient mice. **b** Expression of CTLA-4 in CD4^+^FoxP3^+^ Treg cells from spleens in WT and *Gpr174*-deficient mice. **c** Expression of IL-10 in CD4^+^FoxP3^+^ Treg cells from spleens in WT mice and *Gpr174*-deficient mice. Data are representative of three independent experiments (*n* = 3 mice/group). Data are shown as mean ± S.D. **P < 0.01

Knockout of *Gpr174* did not significantly affect the percentages of Treg cells (CD4^+^CD25^+^FoxP3^+^ T cells), CD4^+^ T cells and CD8^+^ T cells (Supplementary Fig. 3-4) and B cells (data not shown) in peripheral immune organs. However, *Gpr174*-deficient mice presented more Treg cells in thymus (Supplementary Fig. 4). Then we further analyzed the mRNA expression levels of immune-related genes in Treg cells from WT and *Gpr174*-deficient mice. We found that mRNA levels of *Ctla-4*, *programmed cell death 1* (*Pdcd-1*), and *IL-10* were elevated in Treg cells from *Gpr174*-deficient mice in comparison with that from WT mice, whereas mRNA levels of *forkhead box p3* (*Foxp3*), *Cd25*, and *transforming growth factor beta 1* (*Tgfβ1*) mRNA were similar (Fig. 2a). In addition, the expression levels of intracellular CTLA-4 and IL-10 in Treg cells were higher in *Gpr174*-deficient mice than that in WT mice using flow cytometry (Fig. 2b, c and Supplementary Fig. 5). However, the expression levels of latency-associated peptide (LAP) and PD-1 showed no differences in Treg cells from WT and *Gpr174*-deficient mice (Supplementary Figs. 6–7). The changed expression levels of regulatory molecules in *Gpr174*-deficient Treg cells indicated that GPR174 may limit the suppressive function of Treg cells.

### *Gpr174*-deficient Treg cells attenuated LPS-induced systemic inflammatory response

**Fig. 3:**
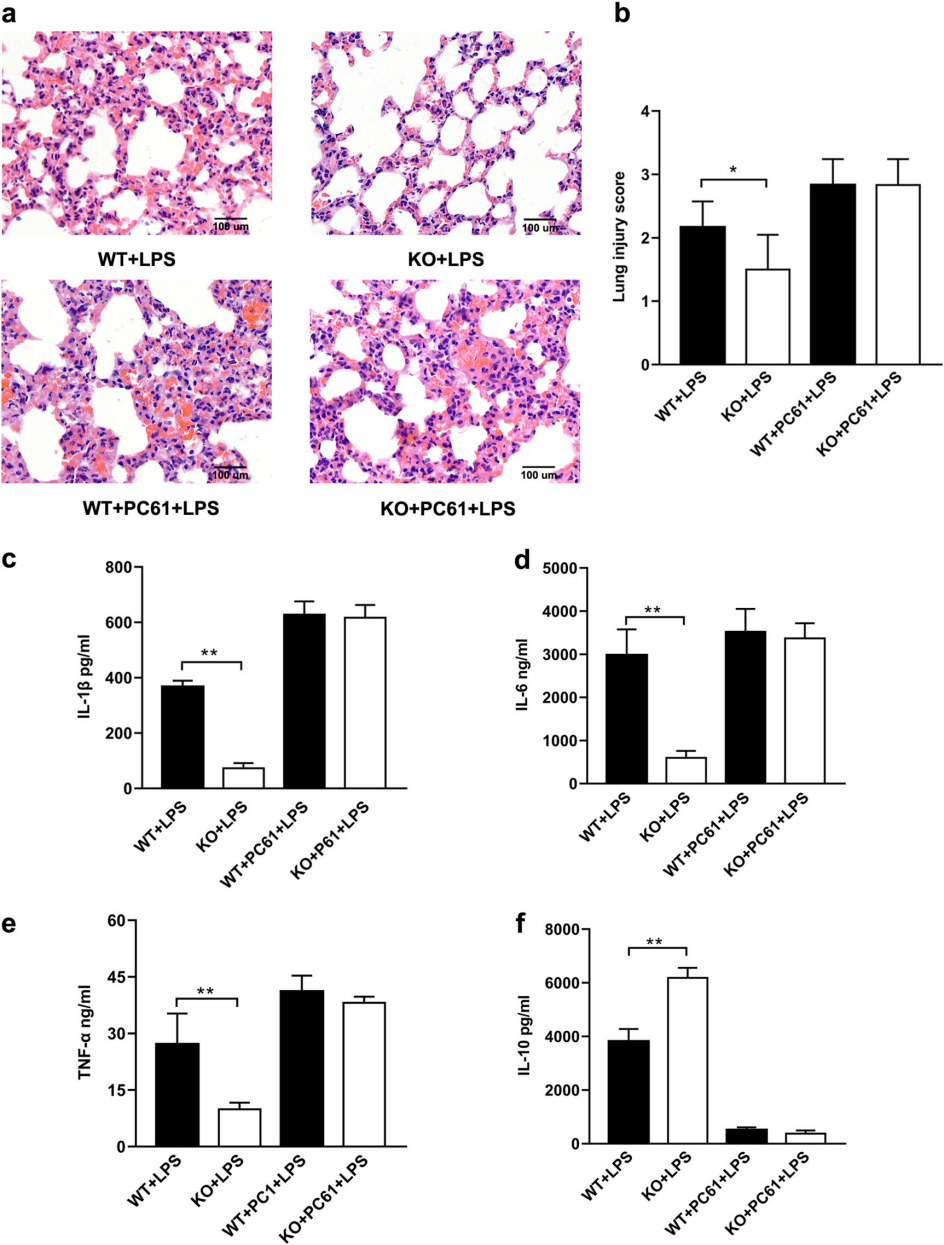
Depletion of *Gpr174*-deficient Treg cells aggravated inflammation response induced lung injury in septic mice. **a** H& E staining of lung tissues from LPS-treated WT and *Gpr174*-deficient mice which received PBS or PC61 treatment 3 days before. **b** Lung injury scores. **c**–**f** Serum cytokine concentrations measured from LPS-treated WT and *Gpr174*-deficient mice which received PC61 or PBS-treatment 3 days before. Data are representative of three independent experiments (*n* = 3 mice/group). Data are shown as mean ± S.D. *P < 0.05; **P < 0.01

Our results showed that knockout of *Gpr174* alleviated LPS induced-lung injuries (Fig. 3a, b) and pro-inflammatory cytokines levels (IL-1β, IL-6 and TNF-α) (Fig. 3c–e). We performed Treg cell depletion study through the injection of PC61 mAb (anti-murine CD25 rat IgG1) in WT and *Gpr174*-deficient mice (Supplementary Fig. 8a)^21^. Depletion of Treg cells aggravated lung injuries (Fig. 3a, b) and increased serum levels of pro-inflammatory cytokines (IL-1β, IL-6, and TNF-α) (Fig. 3c–e) in both septic WT and *Gpr174*-deficient mice. Meanwhile, depletion of Treg cells in WT and *Gpr174*-deficient mice resulted in low levels of IL-10 (Fig. 3f), which plays an important role in attenuating sepsis induced tissue damage. However, no difference of pro- or anti-inflammatory cytokines was found between WT and *Gpr174*-deficient mice after depletion of Treg cells (Fig. 3c–f).

**Fig. 4:**
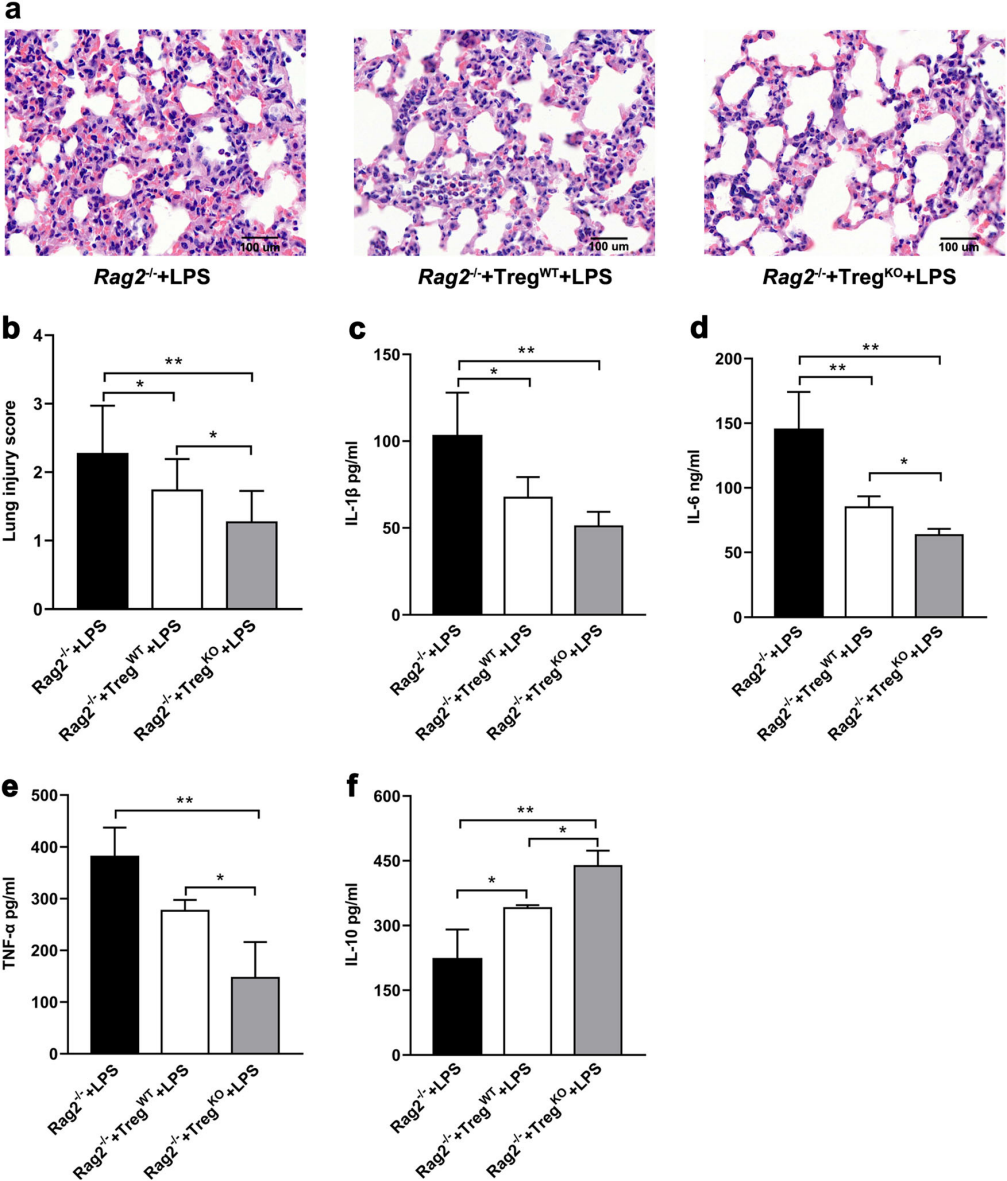
Adoptive transfer of *Gpr174*-deficient Treg cells alleviated LPS-induced lung injury. **a** H& E staining of lung tissues from LPS-treated B6.*Rag2*^−/−^ mice, and B6.*Rag2*^*−/−*^ mice which received WT and *Gpr174*-deficient mouse Treg cells adoptive transfers 16 h before LPS injection. **b** Lung injury scores. **c**–**f** Serum cytokine concentrations measured from LPS-treated B6.*Rag2*^−/−^ mice, and B6.*Rag2*^−/−^ mice which received WT and *Gpr174*-deficient mouse Treg cells adoptive transfers 16 h before LPS injection. Data are representative of three independent experiments (*n* = 3 mice/group). Data are shown as mean ± S.D. *P < 0.05; **P < 0.01

To further explore the protective function of *Gpr174*-deficient Treg cells in sepsis, we performed an adoptive transfer study^22^. CD4^+^CD25^+^ T cells separated from spleens were intravenously injected into *Rag2*^−/−^ mice at 1 × 10^6^ cells before LPS intraperitoneal injection. To confirm that the exogenous spleen-derived Treg cells could migrate to the lung of recipient mice, we stained lung tissue with FoxP3 monoclonal antibody and counted the positive cells. WT and *Gpr174*-deficient mouse Treg cell numbers at 16h after adoptive transfer in the lungs showed no difference in the recipient mice (WT mouse Treg cells vs. *GPR174*-deficient mouse Treg cells: 32.4 ± 5.86 vs. 27.8 ± 2.99 cells per photograph, 5 photographs from *n* = 3 mice/group, Supplementary Fig. 8b). However, the lung injury appeared mild in *Rag2*^−/−^ mice, which received *Gpr174*-deficient Treg cells (Fig. 4a, b). Sera from mice receiving *Gpr174*-deficient Treg cells contained significantly least IL-1β, IL-6, and TNF-α (Fig. 4c–e), while IL-10 levels were highest among the three groups (Fig. 4f). Taken together, these results suggested that *Gpr174*-deficient Treg cells alleviated lung injury by attenuating pro-inflammatory response in sepsis.

### *Gpr174*-deficient Treg cells limited LPS-induced pro-inflammatory response by modulating macrophage polarization

**Fig. 5:**
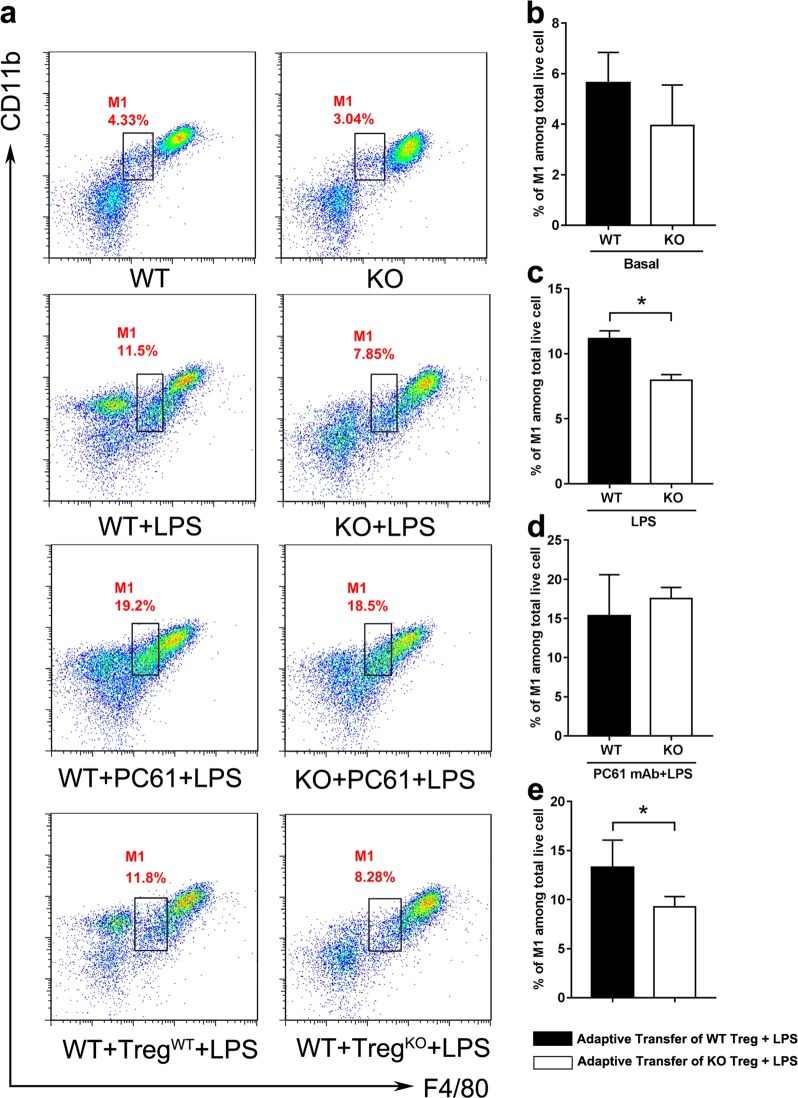
*Gpr174*-deficient Treg cells were associated with lower M1-like peritoneal macrophages in LPS-induced sepsis. Peritoneal cells from PBS-treated or LPS (6 mg/kg) challenged WT and *Gpr174*-deficient mice were analyzed. Peritoneal cells after PC61 injection (200 μg/mouse, 3 days before LPS injection) or adoptive transfer of WT and *Gpr174*-deficient Treg cells (16 h before LPS injection) in LPS induced septic mice were analyzed by flow cytometry. **a** A representative flow cytometry result of M1-like (F4/80^int^CD11b^int^) macrophages were defined within total peritoneal macrophages following co-staining with F4/80 and CD11b. **b**–**c** Quantification of M1-like macrophages extracted from PBS or LPS challenged mice. **d** Quantification of M1-like macrophages extracted from LPS challenged mice after PC61 injection. **e** Quantification of M1-like macrophages extracted from LPS challenged mice after adoptive transfer. Data are representative of three independent experiments (*n* = 3 mice/group). Data are shown as mean ± S.D. *P < 0.05

**Fig. 6:**
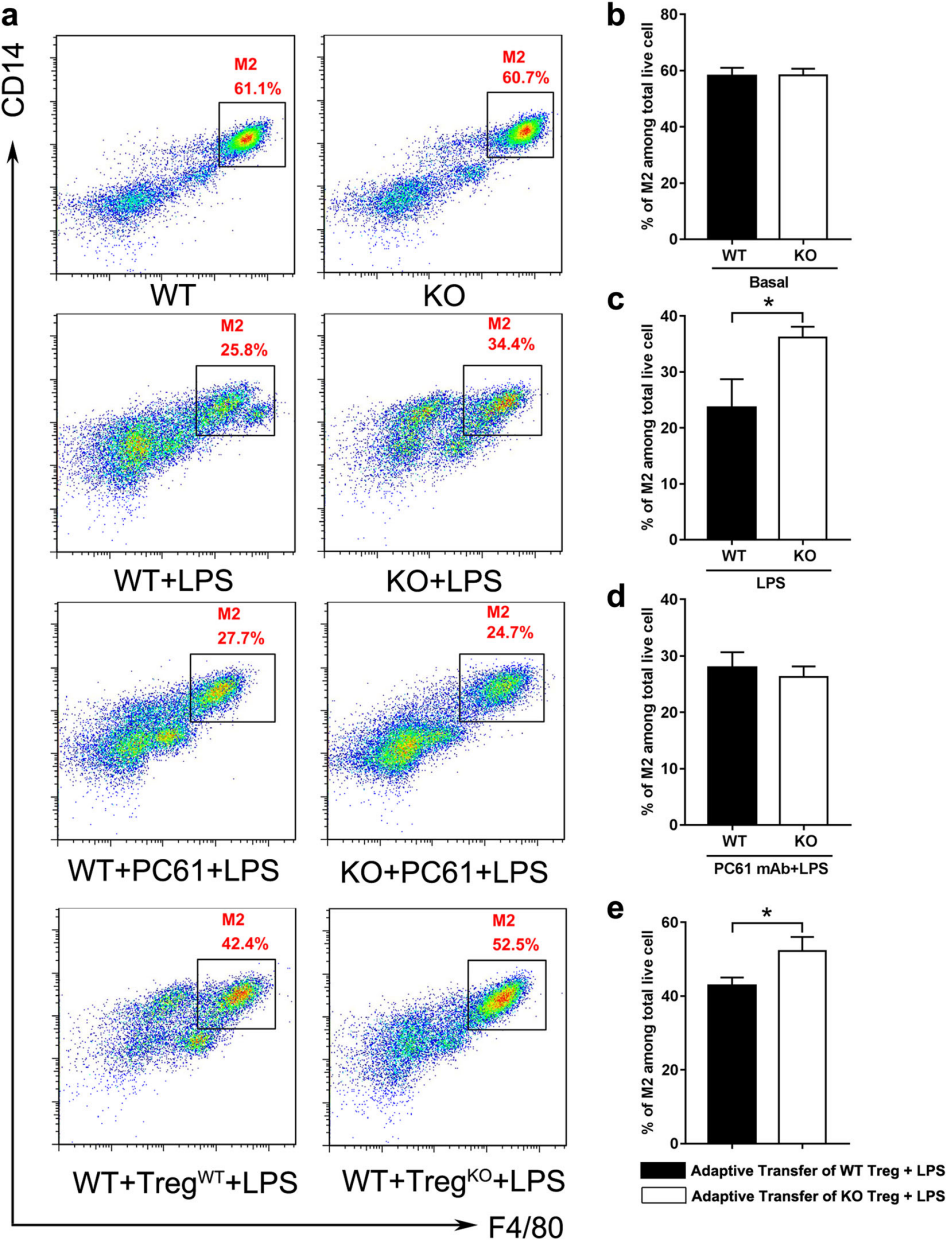
*Gpr174*-deficient Treg cells were associated with a dominance of M2-like macrophages in LPS-induced sepsis. Methods as described in Fig. 5. **a** A representative flow cytometry result of M2-like (F4/80^high^CD14^high^) macrophages were defined within total peritoneal macrophages following co-staining with F4/80 and CD11b. **b**–**c** Quantification of M2-like macrophages extracted from PBS or LPS challenged mice. **d** Quantification of M2-like macrophages extracted from LPS challenged mice after PC61 injection. **e** Quantification of M2-like macrophages extracted from LPS challenged mice after adoptive transfer. Data are representative of three independent experiments (*n* = 3 mice/group). Data are shown as mean ± S.D. *P < 0.05

Treg cells could suppress LPS-induced macrophages/monocytes activation and promote polarization of macrophages toward M2 (M2 or M2-like) macrophages^23,24^. Then, we explored whether *Gpr174*-deficient Treg cells affected the polarization of macrophages in the process of sepsis. In the physiological condition, the percentages of M1 (F4/80^int^CD11b^int^) (Fig. 5a, b) and M2 (F4/80^hi^CD14^hi^) (Fig. 6a, b) macrophages in the peritoneal cavities of WT and *Gpr174*-deficient mice showed no difference. WT mice showed a highly significant increase of M1 macrophages (Fig. 5a, c) and a significant decrease of M2 macrophages (Fig. 6a, c) at 24 h after LPS injection. However, the percentage of M1 macrophages (Fig. 5a, c) remained relative lower and the percentage of M2 macrophages (Fig. 6a, c) remained relative higher in *Gpr174*-deficient mice after LPS administration. The depletion of CD4^+^CD25^+^ T cells resulted in a decrease of M2 macrophages in both WT and *Gpr174*-deficient septic mice (Fig. 6a, d). Adoptively transferred Treg cells from WT and *Gpr174*-deficient mice to recipient mice both increased M2 macrophages of septic recipient mice (Fig. 6a, e). However, the presence of *Gpr174*-deficient Treg cells in septic recipient mice resulted in higher M2 macrophage population (Fig. 6a, e).

**Fig. 7:**
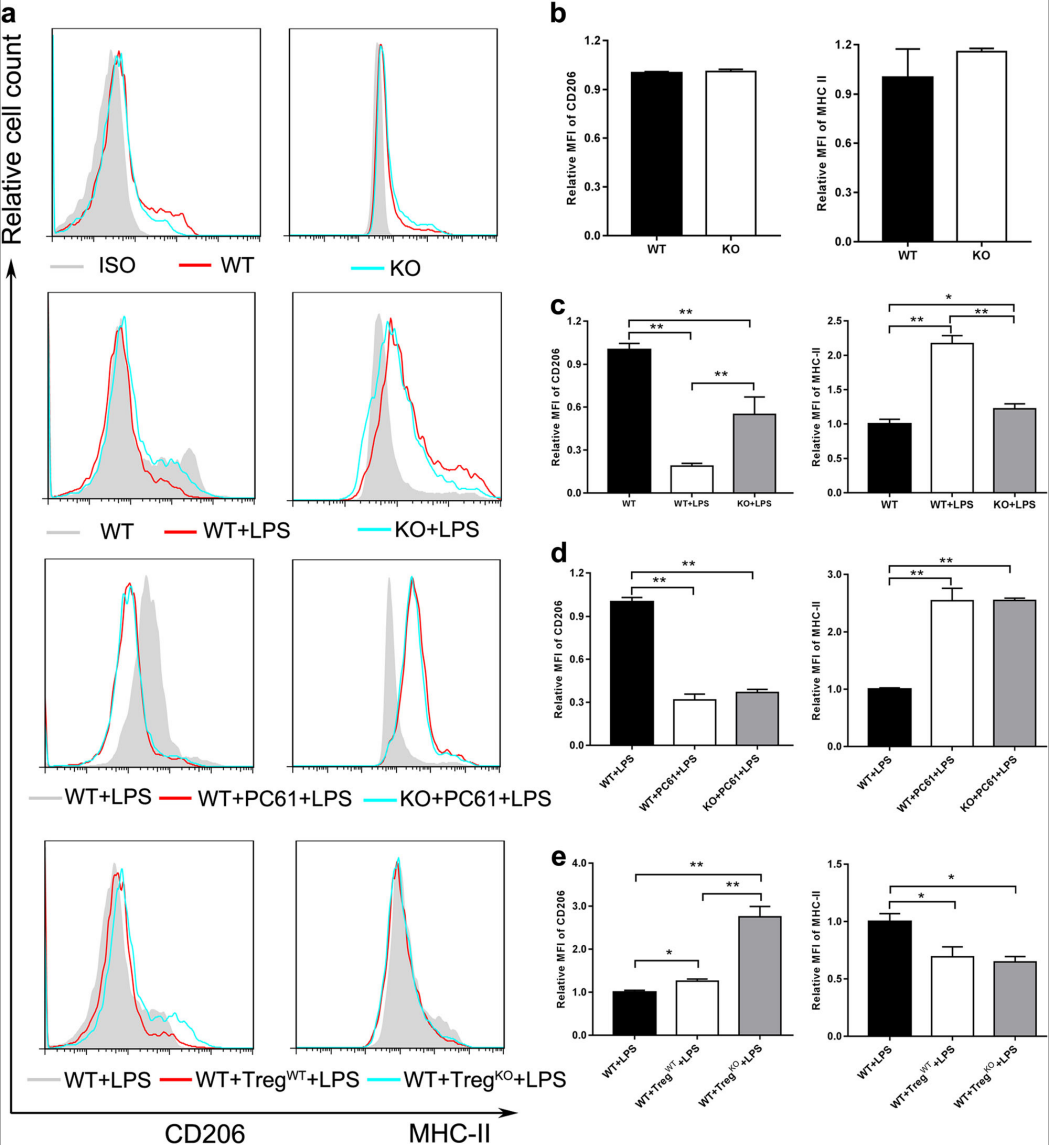
Macrophage phenotypic alterations induced by WT and *Gpr174*-deficient mouse Treg cells. **a** A representative flow cytometry result of F4/80^+^CD11b^+^ macrophage stained with anti-CD206 and MHC-II. Phenotype of macrophage characteristics of septic mice that received none, PC61 injection (200 μg/mouse, 3 days before LPS injection) and Treg cells adaptive transfer (16 h before LPS injection). **b** Quantification of CD206 and MHC-II in macrophages of physiological status mice. **c** Quantification of CD206 and MHC-II in macrophages of LPS challenged mice (24 h). **d** Quantification of CD206 and MHC-II in macrophages of LPS challenged (24 h) mice that received PC61 injection. **e** Quantification of CD206 and MHC-II in macrophages of LPS challenged (24 h) mice after Treg cells adoptive transfer. Data are representative of three independent experiments (*n* = 3 mice/group). Data are  shown as mean ± S.D. *P < 0.05; **P < 0.01

We further used CD206 and MHC-II as the markers of macrophage polarization, since CD206 expression is up-regulated in anti-inflammatory macrophages (M2 macrophages) and MHC-II expression is up-regulated in pro-inflammatory macrophages (M1 macrophages). F4/80^+^CD11b^+^ macrophages displayed the same CD206 and MHC-II levels in WT mice as *Gpr174*-deficient mice (Fig. 7a, b); however, these cells displayed higher CD206 level and lower MHC-II level in *Gpr174*-deficient septic mice than WT septic mice (Fig. 7a, c). These results suggested that more anti-inflammatory macrophages (M2 macrophages) and less pro-inflammatory macrophages (M1 macrophages) were induced after LPS application in *Gpr174*-deficient mice. Moreover, macrophages displayed lower level of CD206 and higher level of MHC-II in both septic WT mice and *Gpr174*-deficient mice after PC61 mAb injection compared to WT mice which only received LPS; whereas, CD206 and MHC-II expressions showed no difference between WT septic mice and *GPR174*-deficient septic mice which received PC61 mAb (Fig. 7a, d). In addition, macrophages in *Gpr174*-deficient Treg cell recipient mice displayed higher level of CD206 compared to that in WT Treg cell recipient mice (Fig. 7a, e). Taken together, these results indicated that *Gpr174*-deficient Treg cells reduced the LPS-induced acute pro-inflammatory response *via* promoting the polarization of macrophages toward M2 cells.

### *Gpr174*-deficient Treg cells restrained LPS-induced inflammatory response in macrophages via IL-10 and contact inhibition-dependent pathways

**Fig. 8:**
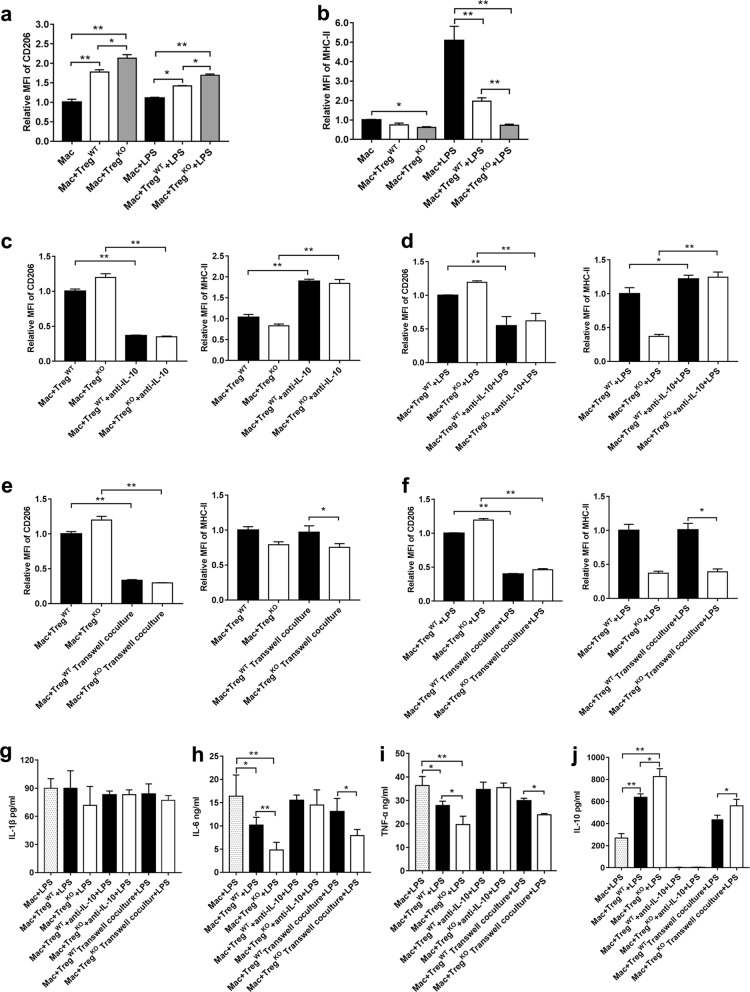
IL-10 and cell-cell contact related suppressive functions of *Gpr174*-deficient Treg cells affected LPS induced-macrophage polarization and activation. **a**–**b** Expression of CD206 and MHC-II on macrophages and LPS stimulated macrophages (24 h) after co-cultured with WT and *Gpr174*-deficient mouse Treg cells (40 h). **c**–**d** Expression of CD206 and MHC-II on macrophages and LPS stimulated macrophages (24 h) after co-cultured with WT and *Gpr174*-deficient mouse Treg cells in the presence of IL-10 antibody (10 μg/ml, 40 h). **e**–**f** Expression of CD206 and MHC-II on macrophages and LPS stimulated macrophages (24h) after co-cultured with WT and *Gpr174*-deficient mouse Treg cells in the Transwell co-cultured system (macrophages in the under layer, Treg cells on the upper layer, 40 h). **g**–**j** Expressions of pro-inflammatory cytokines (IL-1β, IL-6, TNF-α, and IL-10) in LPS stimulated macrophages in the indicated conditions. Data are representative of two independent experiments (n = 6 wells/group). Data are shown  as mean ± S.D. *P < 0.05, **P < 0.01

To further identify the anti-inflammatory activity of *Gpr174*-deficient Treg cells, we co-cultured WT mouse Treg cells or *Gpr174*-deficient Treg cells with WT mouse macrophages in vitro (Supplementary Fig. 9a–b). Compared with macrophages cultured alone, both WT mouse Treg cells and *Gpr174*-dedicient Treg cells promoted the expression of CD206 on macrophages (Figs. 8a, Supplementary Fig. 9c). As expected, the expression level of CD206 in macrophages co-cultured with *Gpr174*-deficient Treg cells in absence of LPS stimulation was significantly higher in comparison with macrophages co-cultured with WT mouse Treg cells in the same culture condition (Fig. 8a, Supplementary Fig. 9c). Moreover, macrophages co-cultured with *Gpr174*-deficient Treg cells expressed higher CD206 and less MHC-II after LPS stimulation compared to that co-cultured with WT mice Treg cells (Fig. 8a, b, Supplementary Fig. 9c–d), suggesting that the immunosuppressive effect of *Gpr174*-deficient Treg cells is more robust than WT mouse Treg cells. These results further supported that anti-inflammatory macrophage (M2 macrophage) induction was Treg cell-dependent in *Gpr174* deficient mice.

In *Gpr174*-deficient Treg cells, IL-10 (cytokine inhibition pathway) and CTLA-4 (cell-cell contact inhibition pathway) might promote the polarization of macrophages toward M2 type cells. To test this hypothesis, neutralizing antibody against IL-10 and Transwell co-culture system were added to the co-culture^25^. IL-10 antibody down regulated CD206 expression and up regulated MHC-II expression on macrophages co-cultured with both two genotype Treg cells in the absence or presence of LPS (Fig. 8c, d, Supplementary Fig. 9e–f). In Transwell co-culture system, *Gpr174*-deficient Treg cells still significantly decreased MHC-II expression on macrophages in the absence or presence of LPS (Fig. 8e, f, Supplementary Fig. 9g–h).

In addition, compared to WT mouse Treg cells, *Gpr174*-deficient Treg cells significantly inhibited the levels of IL-6 and TNF-α produced by macrophages (Fig. 8h, i). Interestingly, macrophages co-cultured with *Gpr174*-deficient Treg cells produced more IL-10 in comparison with macrophages alone or co-cultured with WT mouse Treg cells (Fig. 8j). IL-10 antibody attenuated the inhibitory effect of *Gpr174*-deficient Treg cells, resulting in up-regulation of pro-inflammatory cytokines (IL-6 and TNF-α) (Fig. 8h, i) in the macrophages. In Transwell co-culture system, *Gpr174*-deficient Treg cells only partially decreased the productions of IL-6 and TNF-α (Fig. 8h, i) and increased the production of IL-10 compared to WT mouse Treg cells (Fig. 8j), indicating that cell-cell contact inhibition-dependent pathway, such as CTLA-4, might be involved in *Gpr174*-deficient Treg cells mediating cytokine-inhibition of macrophages. Thus, *Gpr174*-deficient Treg cells may promote macrophage polarization towards M2 phenotype through IL-10 dependent pathway and at least in part through cell-cell contact pathway such as CTLA-4 dependent pathway.

## Discussion

The major finding of this study was that *Gpr174*-deficient Treg cells controlled and down regulated the pro-inflammatory cytokines in LPS-induced sepsis, and thus leaded to dramatical attenuation of LPS-induced tissue injury. The protective function of *Gpr174*-deficient Treg cells was associated with a striking increased of M2 macrophages in sepsis. In addition, IL-10 cytokine inhibition pathway and cell-contact suppressive function of *Gpr174*-deficient Treg cells might regulate immune response of macrophages by promoting the differentiation of M2 macrophages as well as by inhibiting the differentiation of M1 macrophages. All these results demonstrated that anti-inflammatory response in *Gpr174*-deficient mice might be due to suppression of macrophage activation by Treg cells, which indicated an essential role for GPR174 in regulating Treg cells suppressive function.

In sepsis, the innate immune system provides a first line of defense against invading pathogens by releasing multiple inflammatory cytokines to combat infectious and recruit additional immune-related response^25^. However, excessive systemic host-inflammatory response could cause organ failure by inducing tissue injury. In the current study, using *Gpr174*-deficient mice, we demonstrated that GPR174 could strengthen LPS-induced tissue injury by amplifying cytokine storm in the initial phase of sepsis.

Treg cells play an essential role in autoimmune tolerance and restrain the exaggerated immune activation induced by LPS^7^. They not only suppress adaptive T cell responses^26,27^ but also control pathological process mediated by innate immune^6,28^. Our results showed that *Gpr174*-deficient Treg cells expressed higher levels of molecules with suppressive function. *Gpr174*-deficient Treg cells had higher levels of IL-10 and CTLA-4 compared to Treg cells from WT mice. It is well known that Treg cells could limit inflammatory storm and reduce collateral tissue damage in early phase of sepsis^29^. Adoptive transfer of *Gpr174*-deficient Treg cells to *Rag2*^−/−^ mice protected recipient mice against LPS challenge. So in our LPS-induced sepsis, tolerance to LPS was associated with *Gpr174*-deficient Treg cells. This protection was associated with reduced serum levels of pro-inflammatory cytokines and increased anti-inflammatory cytokine.

GPCR signal was demonstrated participating in the development of Treg cells^19,30^. Our results were inconsistent with previous study that *Gpr174* deficiency significantly increased the frequency of FoxP3^+^CD4^+^ single positive (SP) T cells in mouse thymus where the natural Treg (nTreg) cells develop^19^. However, the frequencies of Treg cells in spleen, mesenteric lymph nodes (MLNs), and blood were not changed in *Gpr174*-deficient mice. In addition, we found that frequencies of CD4^+^ SP and CD8^+^ SP T cells in the thymus as well as frequencies of CD4^+^ T cells and CD8^+^ T cells in the periphery (spleen, MLNs, and blood) were equivalent in WT and *Gpr174*-deficient mice. It could be speculated that GPR174 signaling appeared to intrinsically constrain the development of nTreg in thymus. Our results also demonstrated that GPR174 had a negative function in the suppressive function of mature Treg cells by regulating effector molecules including IL-10 and CTLA-4. PD-1 played an important role in maintenance of Treg cell-mediated suppression^8,31^. Although *Pdcd-1* mRNA level in *Gpr174*-deficient Treg cells was higher than that in WT mouse Treg cells, we did not find increased PD-1 protein in *Gpr174*-deficient Treg cells by FACS. These inconsistencies need further validation and exploration. The mechanisms of Treg cell-mediated immune suppression in maintaining immune homoeostasis have been widely explored. However, regulatory mechanisms of stability and suppressive function of Treg cell remain unclear. Future studies are required to clarify the underlying mechanism of GPR174 signal on the expression of IL-10 and CTLA-4 in Treg cells.

Accumulating evidences have suggested that macrophage plays a critical role in microbial clearance and inflammatory response to endotoxin during sepsis^3^. Activated macrophages are generally categorized as pro-inflammatory M1 macrophages and anti-inflammatory M2 macrophages based on their phenotype and function. Concentrations of M1 type cytokines were significantly higher in septic shock patients than that in severe sepsis patients^32^. M2 macrophages alleviate LPS-induced pro-inflammatory and tissue damage in sepsis^3^. We found that deletion of *Gpr174* facilitated the phenotype shift from M1 to M2 macrophages. Knockout of *Gpr174* dampened the M1-associated cytokines (IL-1β, IL-6 and TNF-α) secretion and MHC-II expression. On the other hand, *Gpr174* deficiency increased M2-associated cytokine (IL-10) secretion and CD206 expression. We also found that *Gpr174*-deficient Treg cells could promote polarization of macrophages toward anti-inflammatory M2 macrophages by selectively Treg cells depletion or adoptive transfer. IL-10 and cell-cell contact pathway of Treg cells could promote the differentiation of M2 macrophages^23,24,33^. Macrophage is one kind of innate cells causing the exaggerated cytokines secretion in sepsis. Co-culture Treg cells and macrophages in vitro further verified the function of *Gpr174*-deficient Treg cells acting on macrophages polarization towards M2 profiles by IL-10 and cell-contact pathway.

In conclusion, our results demonstrated that *Gpr174* deficiency plays a protective role in septic mice. *Gpr174*-deficient Treg cells controlled macrophage polarization via IL-10 dependent and cell-cell contact dependent pathway in LPS-induced sepsis. Our data also found that GPR174 regulated the function of Treg cells involved in the pro- and anti-inflammatory cytokine secretions during sepsis and highlighted GPR174 as a potential therapeutic target for clinical intervention to manage sepsis.

## Materials and methods

### Mice

*Gpr174* KO mice were generated using a homologous recombination method and housed in specific pathogen-free condition with a 12 h-light/12 h-dark cycle per day at 22–24 °C. Age-matched male KO mice and male littermate WT mice (8–12 weeks) were used for all experiments. Animal experiments were approved by Ethics Committee of Laboratory Animal Science, Fudan University (201804001Z).

### LPS and CLP-induced sepsis models

Endotoxemia mouse model was induced by intraperitoneal injection (i.p.) with LPS (10 mg/kg, Escherichia coli 055:B5, Sigma-Aldrich, Darmstadt, Germany). CLP-induced sepsis was generated as described previously^20^. In brief, mouse was anesthetized with 1% pentobarbital and a 1–1.5 cm midline incision was made. About 50% of the cecum was ligated and the cecum was punctured once with a 21-gauge needle. A small amount of feces was extruded from the hole to ensure patency. The abdominal incision was closed by applying sample running sutures. Then, pre-warmed normal saline (50 ml/kg) was injected subcutaneously. The survival was monitored (*n* = 20 per group). At the end point of experiments, peritoneal macrophages, and lung tissues were collected for further analysis.

### Flow cytometry

Cell suspensions were stained with combinations of following monoclonal fluorescently conjugated antibodies: TCRβ eFluor^®^ 450 (H57-597, eBioscience, San Diego, CA, USA), CD4 FITC (GK1.5, eBioscience), CD8a PerCP-Cyanine5.5 (53–6.7, eBioscience), CD25 APC (PC61.5, eBioscience), FoxP3 PE (NRRF-30, eBioscience), CD45R/B220 APC-Cyanine7 (RA3-6B2, Biolegend, San Diego, CA, USA), IgD Brilliant violet 650™ (11-26c.2a, Biolegend), CD11b Alexa Fluor^®^ 700 (M1/70, Biolegend), F4/80 PE (BM8, Biolegend), PD-1 PE-eFluor™ 610 (J43, eBioscience), CTLA-4 PerCP-Cyanine5.5 (UC10-4B9, Biolegend), IL-10 PerCP-Cyanine5.5 (JES5-16E3, Biolegend), LAP PerCP-Cyanine5.5 (TWT-16B4, Biolegend), CD14 FITC (Sa2-8, eBioscience), CD206 APC (MR6F3, eBioscience), MHC-II e Fluor^®^ 450 (M5/114.15.2, eBioscience). To stain for intracellular murine antigens, cells were first stained for surface antigens, then fixed and permeabilized with intracellular fixation and permeabilization buffer kit (eBioscience) according to the manufacturer’s recommendation. For intracellular IL-10 staining, cells were incubated with PMA (50 ng/ml) and ionomycin (1000 ng/ml) for 2 h, then incubated with Brefeldin A (10.6 μM) and Monensin (2 μM) for 3 h. Data were acquired using BD Fortessa X20 (BD Bioscences, San Diego, CA, USA) and analyzed with FlowJo 7.6 software.

### RNA isolation and real-time quantitative PCR

Total RNA from different immune cells was isolated using Trizol reagent (Life Technologies, Carlsbad, CA, USA), according to the manufacturer’s instructions. cDNA was generated using Prime Script™ reagent kit (Takara, Dalian, China). Real-time quantitative PCR was performed using SYBR^®^ Premix Ex Taq™ II on either 7500 Real time PCR system (Applied Biosystems, Carlsbad, CA, USA) or CFX-Connect Real time PCR system (Bio-Rad Laboratories, Berkeley, CA, USA). All data were normalized to *Hprt*, analyzed using comparative CT method and presented as the fold-increased over CD8^+^ T cells control.

### CD4^+^CD25^+^ T cell purification and adoptive transfer

T cells were purified from spleens of WT and *Gpr174* KO mice, mechanically disrupted over a 75 μm filter, and subjected to magnetic bead enrichment for CD4^+^CD25^+^ T cells using CD4^+^CD25^+^ Treg isolation kit (Miltenyi Biotec, Bergisch Gladbach, Germany). Enriched CD4^+^CD25^+^ T cells were counted and resuspended in PBS. A cell suspension containing 1 × 10^6^ per 100 μl PBS was injected into C57BL/6 *Rag2*^−/−^ mouse via tail vein.

### Histological analysis

Lung tissue samples were fixed in 4% paraformaldehyde and tissue sections were stained with H&E stain. Lung damage examination was analyzed by tissue injury scoring system, as described previously^34^.

### Cytokine analysis

Serum cytokine levels of IL-1β, IL-6, TNF-α, and IL-10 were measured by ELISA kit (eBioscience) according to the manufacturer’s protocol.

### Differentiation of bone marrow-derived macrophages

Bone marrow cells were isolated from femurs and tibias of WT and *Gpr174* KO mice. Differentiation of Bone Marrow-Derived Macrophages was induced by culturing bone marrow cells in MEM-F12 medium supplemented with 10% FBS and macrophage-colony stimulating factor (20 ng/ml M-CSF, proteintech, Rosemont, PA, USA). Bone Marrow-Derived Macrophages were cultured for 7 days with one renewal of culture medium on the third day.

### In vitro macrophage suppression assay

WT mice Marrow-Derived macrophages were resuspended in RPMI containing 10% FBS. CD4^+^CD25^+^ Treg cells and Marrow-Derived macrophages were seeded on 24-well plate in a 1:2 ratio. Macrophages were cultured alone or co-cultured with Treg cells for 40 h in the presence of 0.4 μg/ml anti-CD3 mAb (UCHT1, eBioscience), then were stimulated with 100 ng/ml LPS for 24 h. Supernatants were harvested for ELISA assay. Macrophages were digested with 0.25% trypsin for flow cytometry.

### Statistical analysis

SPSS (version 19.0, SPSS Inc., Chicago, IL, USA) software was used to analyze data by two-tailed unpaired student’s *t* test or one-way analysis of variance test. Data are presented as mean ± SD. *P* < 0.05 was considered statistically significant (*P < 0.05; **P < 0.01).

## Supplementary information

Supplementary Figure Legends

Supplementary Figure 1

Supplementary Figure 2

Supplementary Figure 3

Supplementary Figure 4

Supplementary Figure 5

Supplementary Figure 6

Supplementary Figure 7

Supplementary Figure 8

Supplementary Figure 9
