# Supplementary material for: Gpr174-deficient regulatory T cells decrease cytokine storm in septic mice
Source: Cell Death Dis. 2019 Mar 8;10(3):233. doi: 10.1038/s41419-019-1462-z (PMC6408576; doi:10.1038/s41419-019-1462-z)
Supplement: Supplementary file 1 — Supplementary Figure Legends [file 41419_2019_1462_MOESM1_ESM.docx]

**Supplementary Figure Legends**

**Supplementary Figure 1 Targeted** **disruption of *Gpr174* in mice.**

(a) *Gpr174* targeting vector was introduced into 129Sv embryonic stem cells for homologous recombination. The floxed region contained exon 5, which included the coding region for *Gpr174*. Coding region of exon 5 is shown as blank box with filled orange (coding region) and the introns 1-4 are shown as blank boxes. Frt sites franking the *neo* selection marker are shown as triangles and LoxP sites flanking the targeted region are arrows. The mutant mice were on a mixed 129Sv/C57BL/6 background. (b) PCR genotyping of progenies from heterozygous mating WT (+/Y) and homozygous (-/Y) mice were identify by PCR amplification of the fragments specific for either *Gpr174* WT allele (520bp) or the mutant allele (732bp). Primes used in PCR assay for mouse genotyping: Forward, CTAAATATTTGTTTGTACCCACAGC; Reverse1, AAGGAGCAAAGCTGCTATTG; Reverse2, TGTTTGGAAGGACGTTTCAT. (c) Thymuses, spleens and mesenteric lymph nodes from 8 weeks old WT and *Gpr174* KO mice. (d) Body weight of 8 weeks old WT and *Gpr174* KO mice (n = 6 mice/group). (e) Total cell number of spleens in 8 weeks old WT and *Gpr174* KO mice (n = 6 mice/group).

**Supplementary Figure 2** **Expression levels of *Gpr174* mRNA in immune cells.**

CD8^+^ T cells, CD4^+^CD25^-^ T cells, CD4^+^CD25^+^ T cells, CD45R^+^IgD^+^ B cells were isolated form mouse spleen cells by using BD FACSAria II storting system. Peritoneal macrophages were harvested by their ability to adhere to culture-treated plastic. Comparison of *Gpr174* mRNA levels in indicated immune cells of WT mice. Expression levels of *Gpr174* mRNA are presented relative to *Hprt* expression. Data are shown as mean ± S.D. (n = 6 mice/group).

**Supplementary Figure 3 *Gpr174* deficiency did not affect CD4 (SP) T and CD8 (SP) T cell populations in central and peripheral immune organs.**

Flow cytometry of gated TCR^+^CD4^+^ (SP) and TCR^+^CD8^+^ (SP) T cells isolated from WT mice and *Gpr174*-deficient mice in thymus, spleen, mesenteric lymph nodes (MLNs), and blood. Right, percents of CD4^+^ (SP) and CD8^+^ (SP) in TCR^+^ cells. Data are representative of three independent experiments (n = 6 mice/group). Data are shown as mean ± S.D.

**Supplementary Figure 4 GPR174 negatively regulated Treg cell population in thymus.**

Flow cytometry of gated TCR^+^CD4^+^CD25^+^FoxP3^+^T cells isolated from WT mice and *Gpr174*-deficient mice in thymus, spleen, MLNs, and blood. Data are representative of three independent experiments (n = 6 mice/group). Data are shown as mean ± S.D.

**Supplementary Figure 5 Increased expression of CTLA-4 in *Gpr174*-deficient Treg cells.**

Flow cytometry of gated TCR^+^CD4^+^ FoxP3^+^CTLA-4^+^ T cells isolated from WT mice and *Gpr174*-deficient mice. Mean fluorescence intensity (MFI) of CTLA-4 in Treg cells in MLNs, blood, and thymus. Data are representative of three independent experiments (n = 3 mice/group). Data are shown as mean ± S.D. **P* < 0.05.

**Supplementary Figure 6 Expression of LAP in *Gpr174*-deficient Treg cells.**

MFI of LAP in CD4^+^FoxP3^+^ Treg cells. Data are representative of three independent experiments (n = 3 mice/group). Data are shown as mean ± S.D.

**Supplementary Figure 7 *Gpr174* deficiency did not affect the expression of PD-1 in Treg cells.**

MFI of PD-1 in CD4^+^FoxP3^+^Treg cells. Data are representative of three independent experiments (n = 6 mice/group). Data are shown as mean ± S.D.

**Supplementary Figure 8 Depletion and Adoptive transfer of *Gpr174*-deficient Treg cells *in vivo*.**

(a) Mice were intraperitoneally injected with PBS or 200 μg of purified anti-CD25 mAb (PC61). Three days later, the spleen cells were collected. The number indicate the percentage of CD4^+^CD25^+^ cells within CD4^+^ cell population and the percentage of FoxP3^+^ cells with the CD4^+^ cell population. The injection of 200μg of PC61 mAb led to a significant depletion of the CD4^+^CD25^+^ T cells and an approximately 50% depletion of the CD4^+^FoxP3^+^ T cells in spleens compared to the groups injected with phosphate buffered saline (PBS). (b) WT mouse Treg cells or *Gpr174*-deficient Treg cells were transferred to B6.*Rag2*^-/-^ mice via caudal vein. FoxP3 immunohistological staining showed no difference in FoxP3^+^ cell numbers between slides from recipient mice receiving Treg cells of WT or *Gpr174*-deficient mice (n = 3, 5 slides/mouse).

**Supplementary Figure 9** **IL-10 and cell-cell contact suppressive function of *Gpr174*-deficient Treg cells affected LPS-stimulated macrophage polarization.**

(a) The purity of Bone Marrow-Derived Macrophages (> 95%). (b) Typan blue stain of Treg cells isolated form mouse spleen cells by using MACS^®^ Separation (Percent of positive cells < 10%). (c-d) MFI of CD206 and MHC-II on LPS stimulated macrophages (24h) before co-cultured with WT and *Gpr174*-deficient mouse Treg cells (40h). (e-f) MFI of CD206 and MHC-II on LPS stimulated macrophages (24h) before co-cultured with WT and *Gpr174*-deficient mouse Treg cells in the presence of IL-10 antibody (10 μg/ml, 40h). (g-h) Expression of CD206 and MHC-II on LPS stimulated macrophages (24h) before co-cultured with WT mouse and *Gpr174*-deficient mouse Treg cells in the Transwell co-cultured system (macrophages in the under layer, Treg cells on the upper layer, 40h).
