# Supplementary figures and images for: Gpr174-deficient regulatory T cells decrease cytokine storm in septic mice
Source: Cell Death Dis. 2019 Mar 8;10(3):233. doi: 10.1038/s41419-019-1462-z (PMC6408576; doi:10.1038/s41419-019-1462-z)

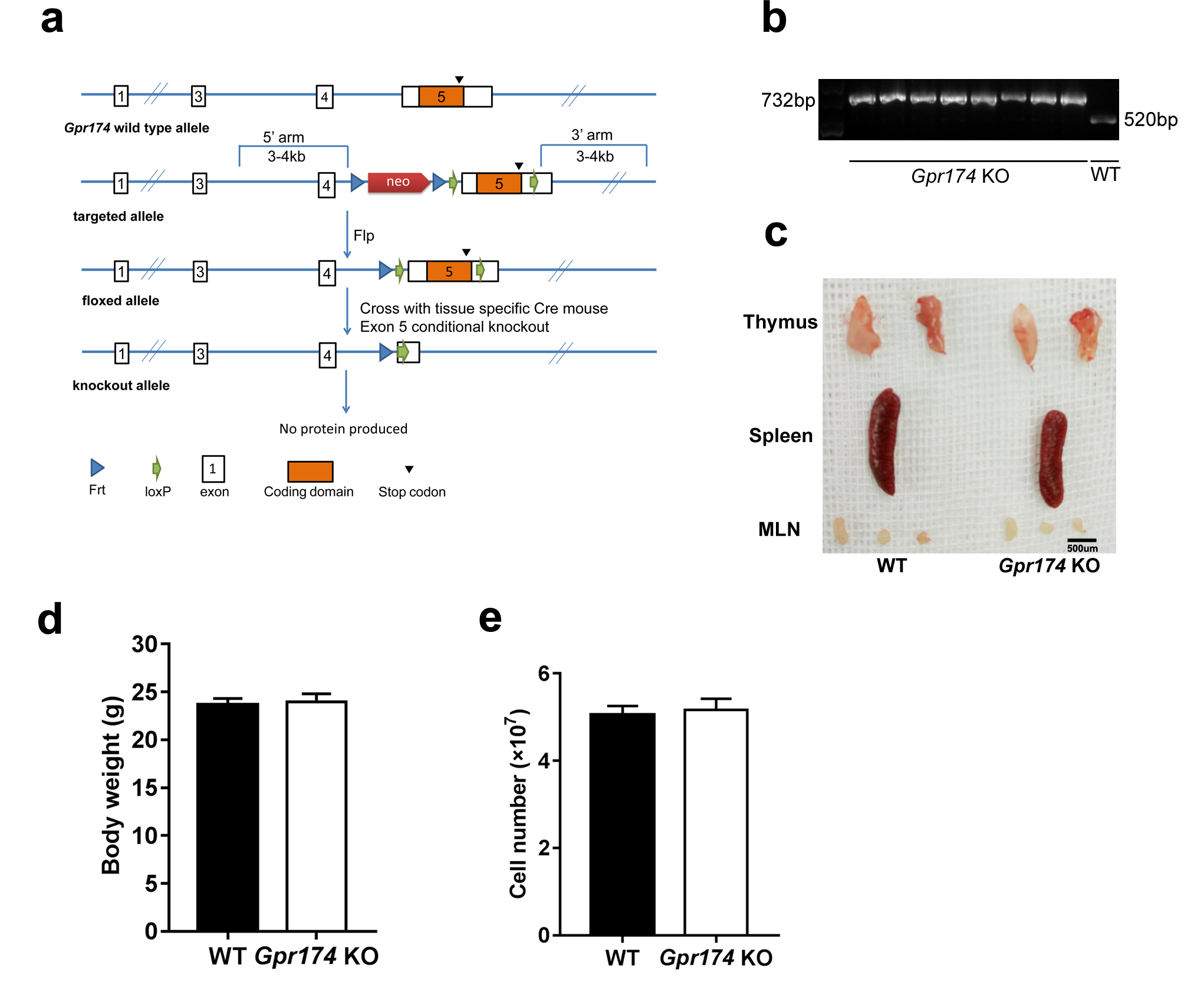

Supplement: Supplementary file 2 — Supplementary Figure 1 [file 41419_2019_1462_MOESM2_ESM.tif]

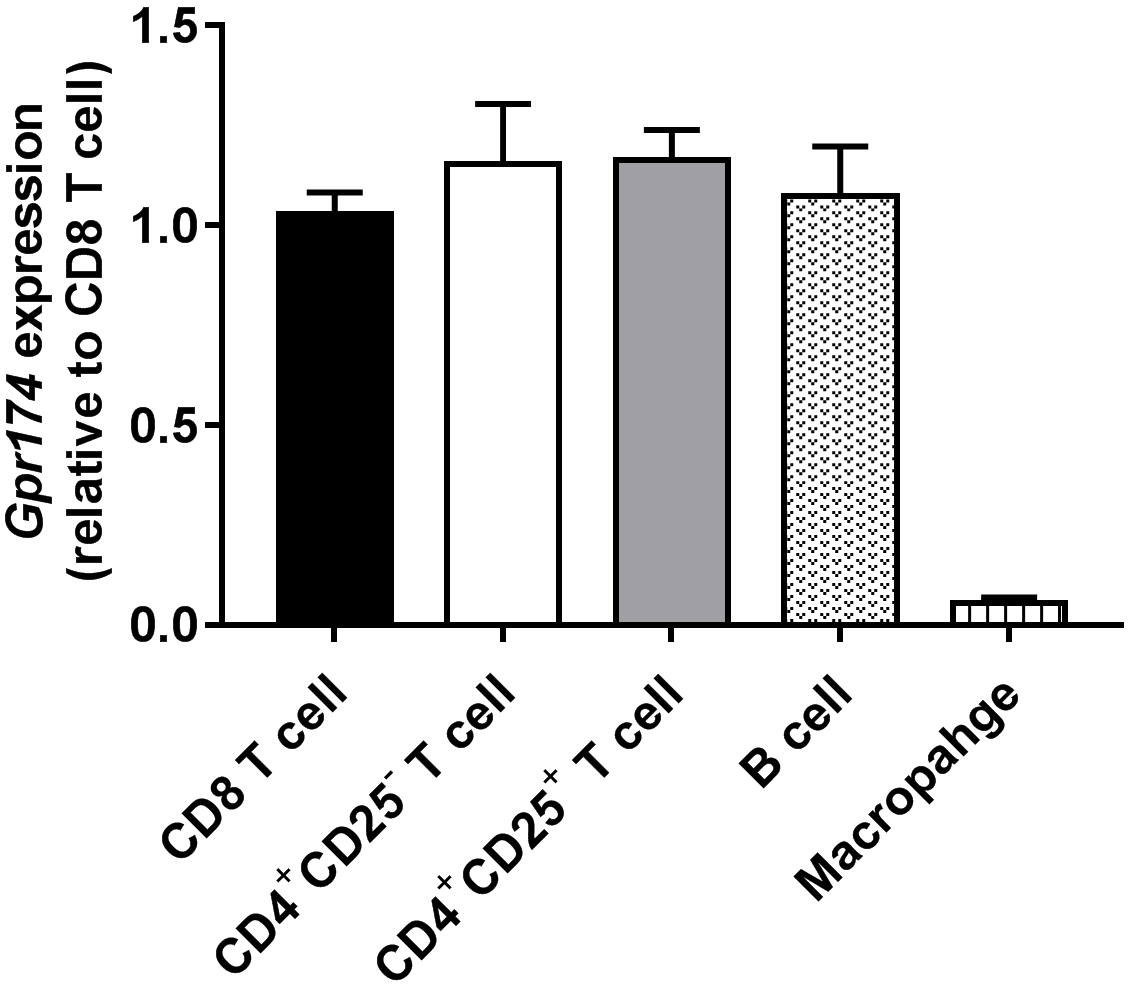

Supplement: Supplementary file 3 — Supplementary Figure 2 [file 41419_2019_1462_MOESM3_ESM.tif]

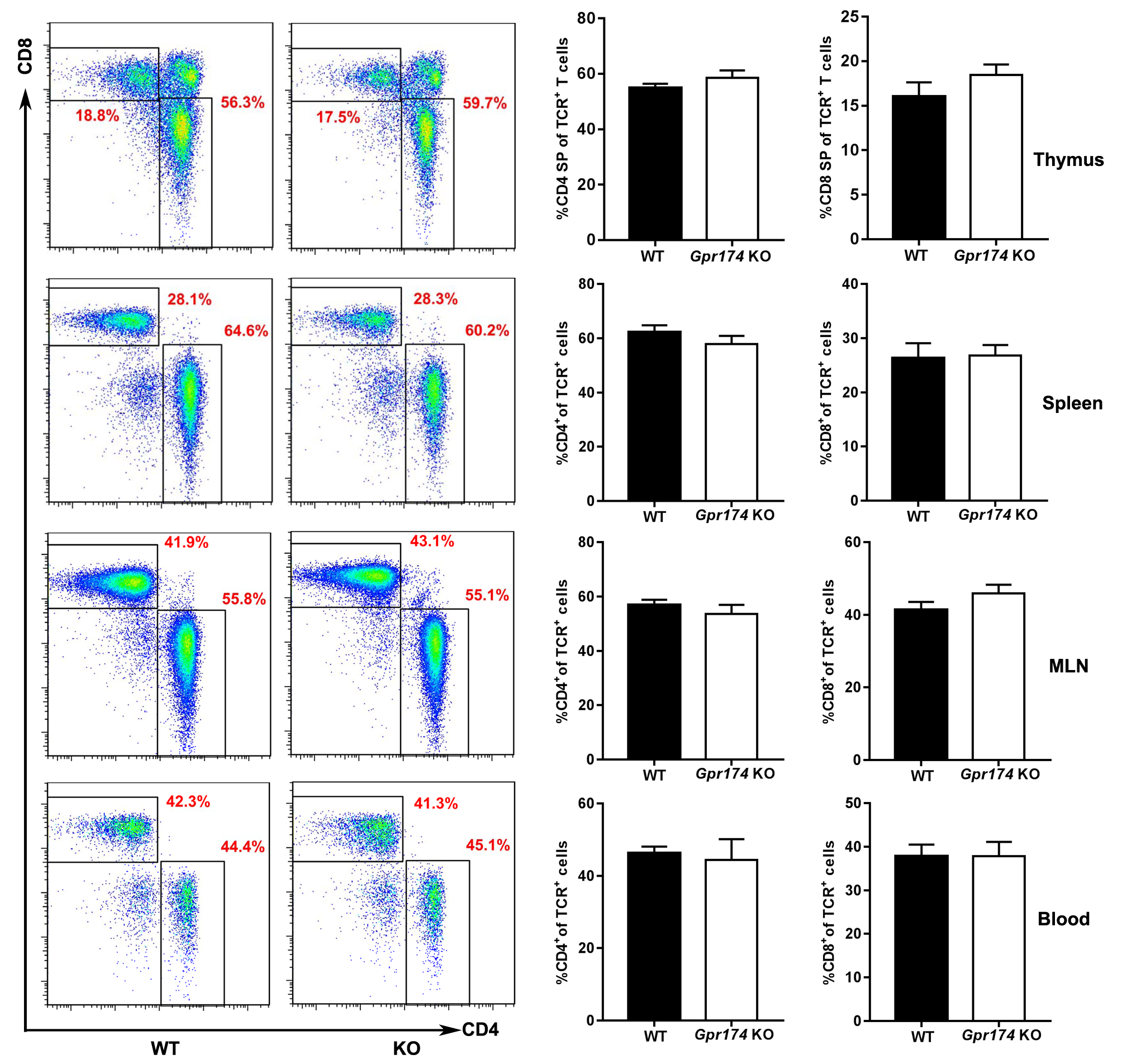

Supplement: Supplementary file 4 — Supplementary Figure 3 [file 41419_2019_1462_MOESM4_ESM.tif]

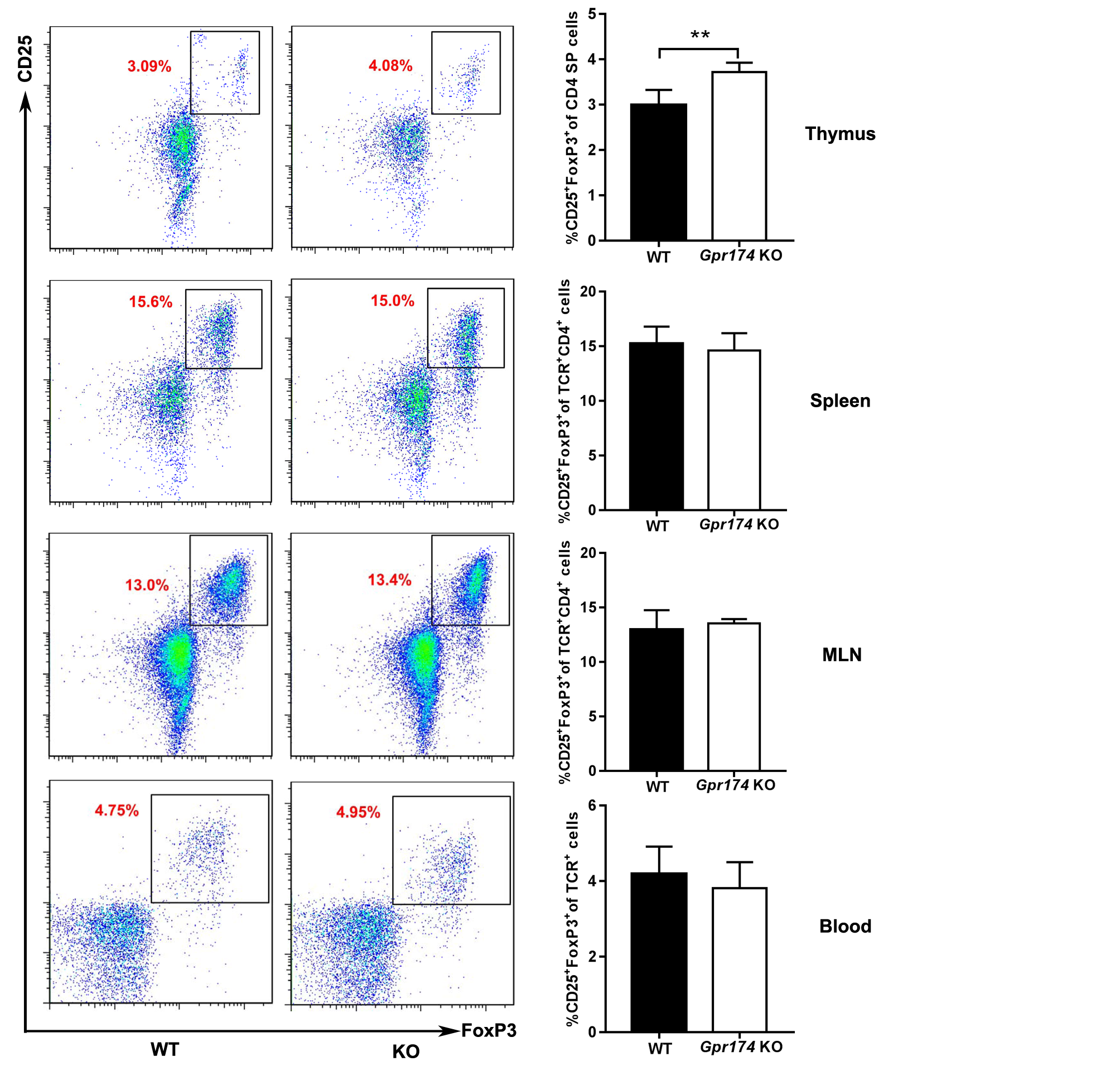

Supplement: Supplementary file 5 — Supplementary Figure 4 [file 41419_2019_1462_MOESM5_ESM.tif]

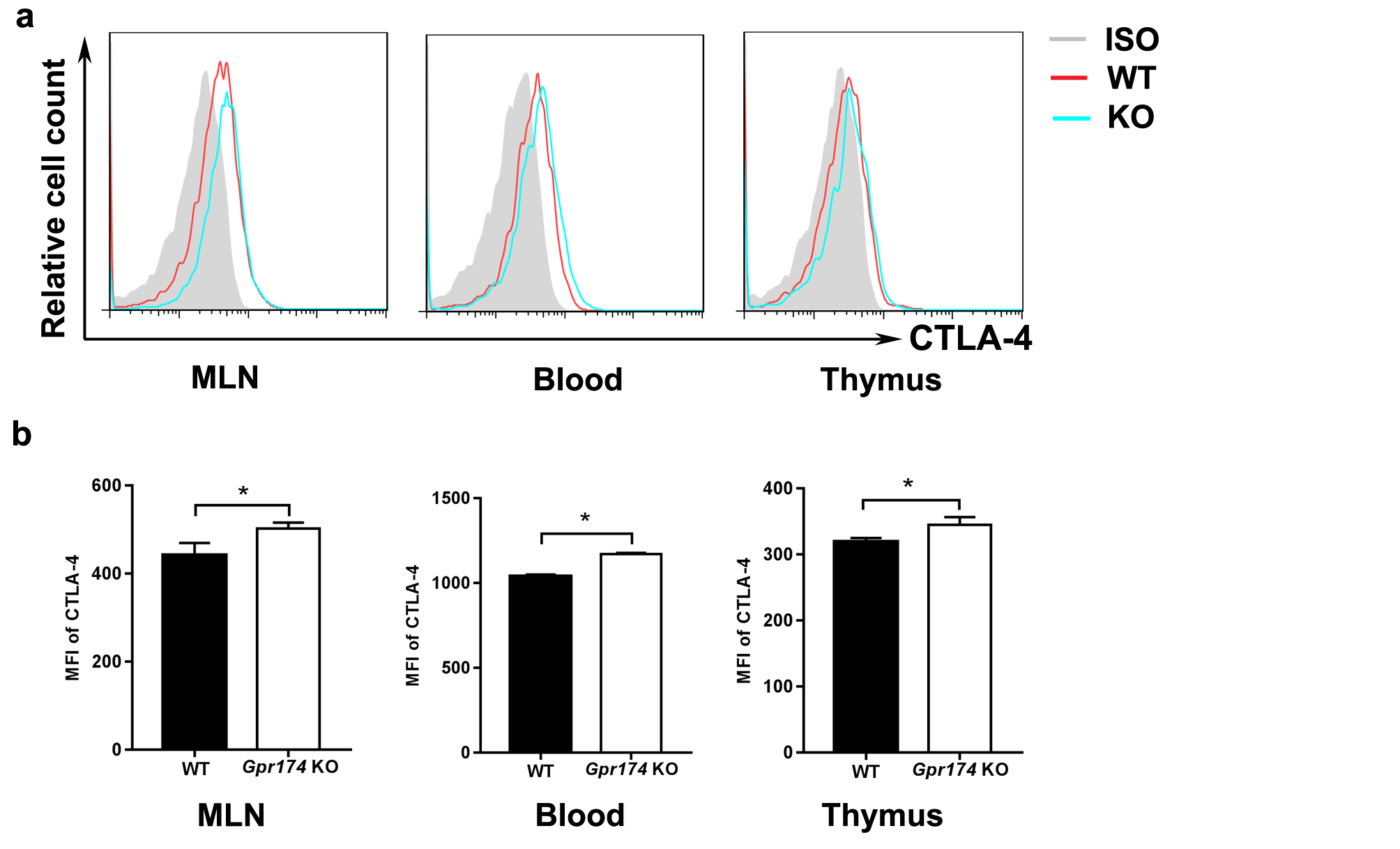

Supplement: Supplementary file 6 — Supplementary Figure 5 [file 41419_2019_1462_MOESM6_ESM.tif]

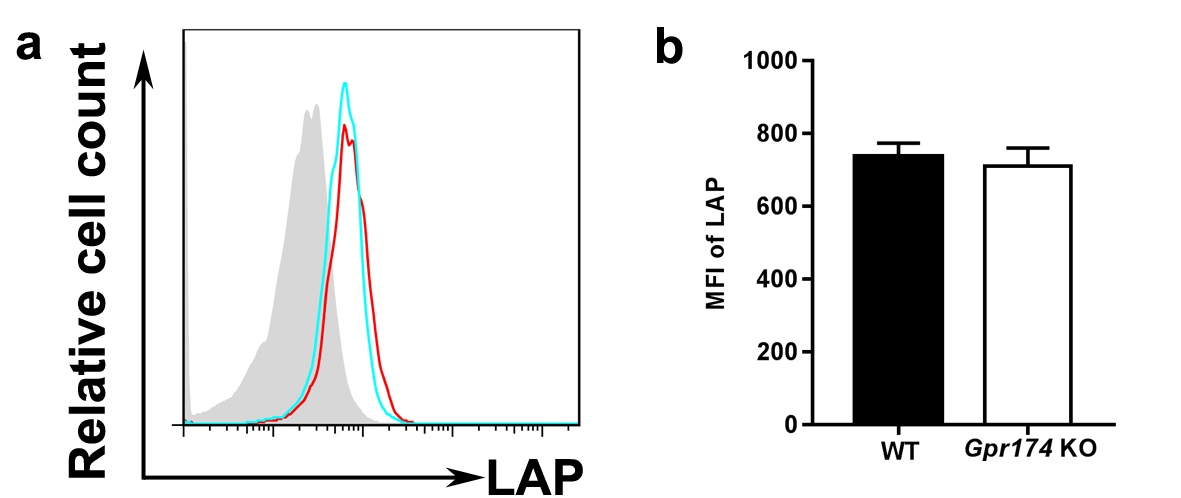

Supplement: Supplementary file 7 — Supplementary Figure 6 [file 41419_2019_1462_MOESM7_ESM.tif]

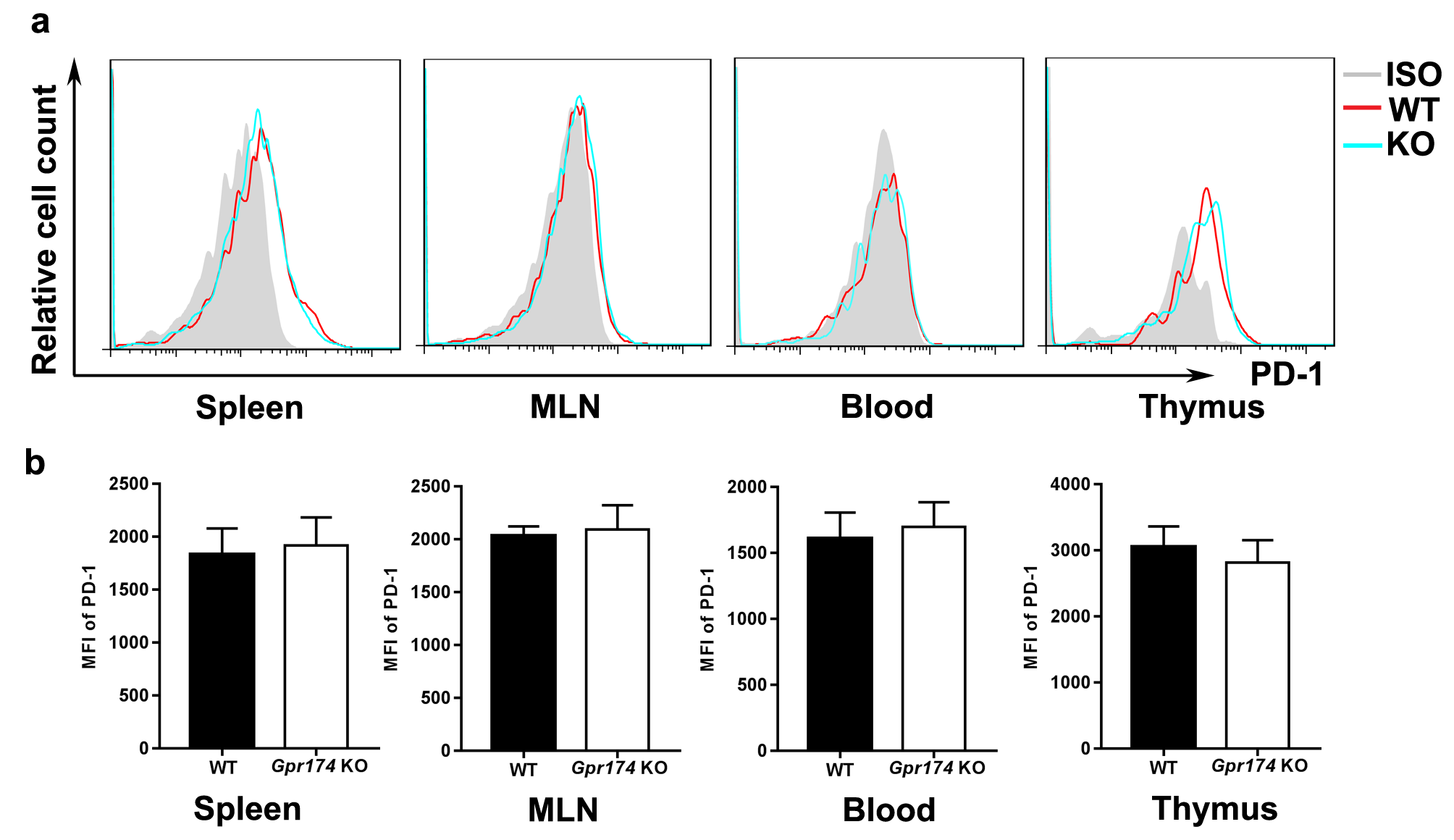

Supplement: Supplementary file 8 — Supplementary Figure 7 [file 41419_2019_1462_MOESM8_ESM.tif]

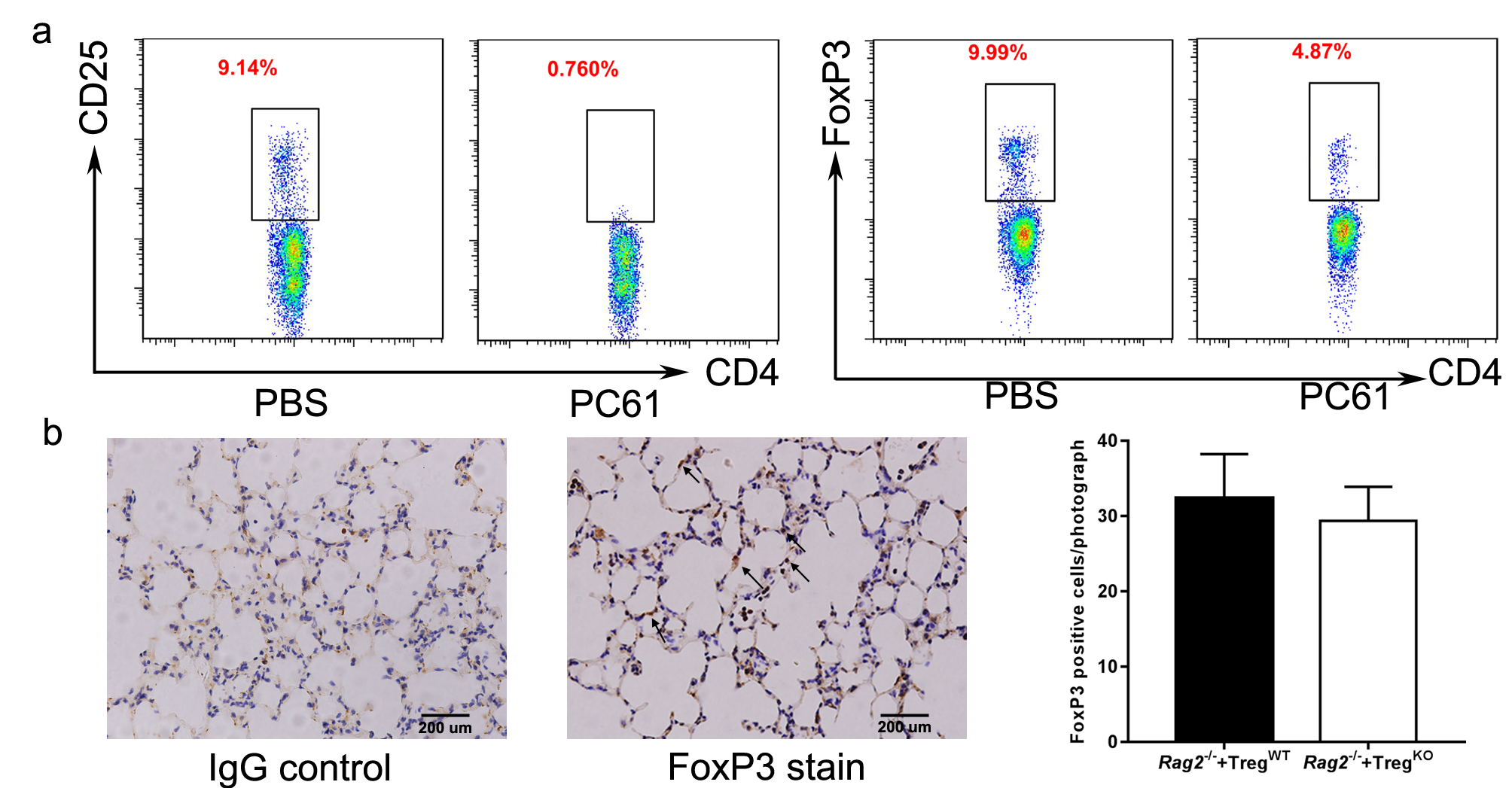

Supplement: Supplementary file 9 — Supplementary Figure 8 [file 41419_2019_1462_MOESM9_ESM.tif]

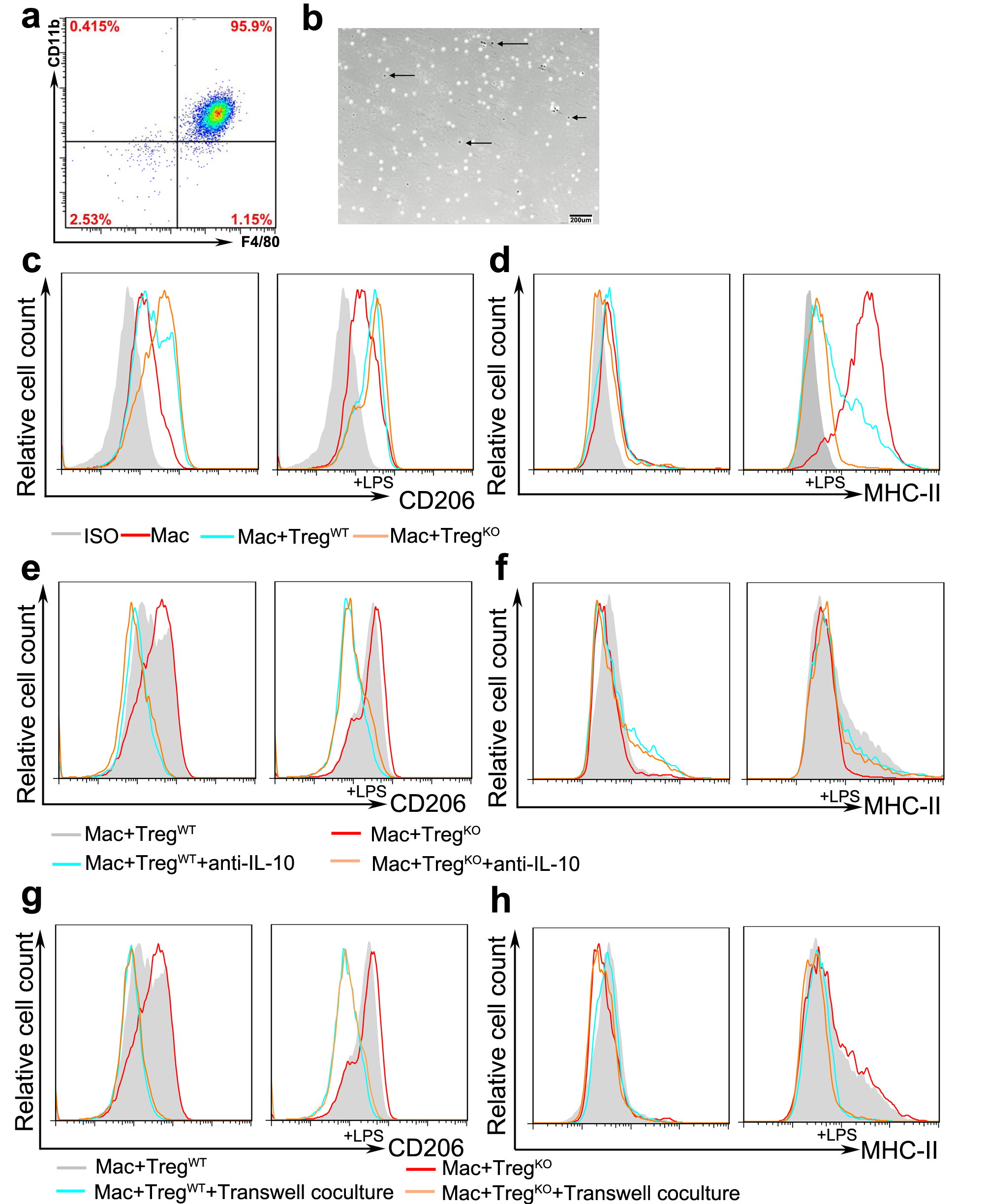

Supplement: Supplementary file 10 — Supplementary Figure 9 [file 41419_2019_1462_MOESM10_ESM.tif]
